# Psoriasis and biological drugs at the time of SARS-CoV-2 infection: a mini review outlining risk of infection, seroprevalence, and safety and efficacy of the BNT162b2 vaccine

**DOI:** 10.3389/fimmu.2024.1354729

**Published:** 2024-01-30

**Authors:** Janosch Railton, Martina Volonté, Eugenio Isoletta, Alice Bonelli, Stefania Barruscotti, Valeria Brazzelli

**Affiliations:** ^1^ Institute of Dermatology, Fondazione IRCCS Policlinico San Matteo, Pavia, Italy; ^2^ Department of Clinical, Surgical, Diagnostic and Pediatric Sciences, Institute of Dermatology, Università degli Studi di Pavia, Pavia, Italy

**Keywords:** psoriasis, frailty, biologic therapy, SARS-CoV-2, BNT162b2 vaccine, targeted immunosuppression

## Abstract

**Objective:**

The aim of this study is to review the life of patients with psoriasis on biologic therapy during the SARS-CoV-2 pandemic and the relevance of frailty within this context, reviewing studies that describe the course and severity of infection in patients with psoriasis on biologics, the seroprevalence of SARS-CoV-2, and the safety and efficacy of the BNT162b2 vaccine in these patients.

**Materials and methods:**

The keywords “Psoriasis,” “Biologics,” “SARS-CoV-2,” “COVID-19,” and “BNT162b2 Vaccine” were used in various combinations on database engines to find relevant articles on this topic.

**Results:**

A total of 36 articles were found, with 20 concerning the course, severity, and seroprevalence of SARS-CoV-2 in patients with psoriasis on biologic therapy and 16 concerning safety and efficacy of BNT162b2 in these patients.

**Discussion:**

Patients with psoriasis on biologic therapy did not have increased seroprevalence compared with the general population, indicating that they were not at an increased risk of SARS-CoV-2 infection compared with the general population. Furthermore, the immunosuppressive action of biologics may be protective, as patients on biologic therapy had better outcomes and less risk of severe infection. The seroconversion rate against SARS-CoV-2 from the BNT162b2 vaccine was similar in both patients with psoriasis on biologics and the general population, indicating that efficacy is not hindered by the biologic therapy. However, the cellular response in population with psoriasis was significantly less intense, and the humoral immune response was weaker than that in the general population, demonstrating that the possibility of tighter vaccination schedules and additional doses may be advantageous in these patients.

## 1 Introduction

Frailty is increasingly becoming more and more relevant within the medical and health care world, due to the increasing life expectancy and the increase in personalized medicine. Recognizing frailty is an important step into further personalizing medicine, so that we can fully assess and care for the patients and their needs, especially concerning protection against disease and treating any current diseases at the same time, weighing out the risks and benefits in each individual. -

The invention of biologic therapy has changed how we treat many diseases, including psoriasis. However, these medications come with a risk, due to secondary immunosuppression, leaving patients vulnerable to infection. The Italian Ministry of Health considers patients on biologic therapy frail. This sparked a discussion about whether or not these patients require extra attention and care during the pandemic, considering that booster doses were recommended to frail patients due to a lower seroconversion and efficacy.

The effect of SARS-CoV-2 on this population will be studied, along with the interaction of biologic therapy on the course of the infection and the risk of increased infections or severe infections within this specific cohort. Finally, the interaction of biologic therapy on the BNT162b2 vaccine in this population will be studied.

## 2 Materials and methods

This review was initiated by searching combinations of the keywords “Psoriasis,” “Biologics,” “Frailty,” “COVID-19,” “SARS-CoV-2,” and “BNT162b2 Vaccine” within various databases such as PubMed and Google Scholar. The abstracts were pre-screened, and the literature was collected.

Following the pre-screening, papers were selected if the met following the criteria:

(1) Literature was written on primary research.
(2) Patients with psoriasis of any form were included.
(3) Patients were on biologic therapy.
(4) The literature was published during the COVID-19 pandemic (from January 2020 to October 2023).

Papers were excluded if they did not follow all of the criteria.

Regarding the literature regarding the BNT162b2 (Pfizer) Vaccine, papers were selected if evidence and results of serologic testing following the efficacy of at least one dose of the vaccine were present.

## 3 Results

Overall, 1,028 articles were found from the initial keyword search; after a brief pre-screening, five articles were discarded because of being in a different language and one article was not available. After screening the titles and abstracts of the 1,022 remaining articles, 998 articles were excluded because of being unrelated to the study. The full text of the 114 remaining articles was reviewed, with 81 articles being screened out because of not following the criteria after analysis.

The remaining 36 articles all met all the inclusion criteria and were chosen for this study. The studies were divided and reviewed into two tables: first, 30 articles were found regarding the course, severity, and seroprevalence of SARS-CoV-2 inpatients with psoriasis (see **Table 1**); and, second, 16 articles regarding the efficacy and safety of the BNT162b2 vaccine in the population with psoriasis on biologics were found (see **Table 2**).

**Table 1:** Course and severity of SARS-CoV-2 infection in patients with psoriasis on biologic therapy.

Author, year	Study design	Type of patients	Number of patients	Results	Notes
**Brazzelli et al., 2020** (1)	Cross-sectional	Psoriatic on biologics	180	No increased incidence or severity in patients with psoriasis on biologic therapy, compared with those on topical therapy; 18.3% of patients with psoriasis reporting at least on symptom due to SARS-CoV-2.	Study preformed via telephone questionnaire due to lockdown restrictions
**Baloghová et al., 2022** (2)	Cross-sectional	Psoriatic	302	No relation between individual comorbidities and number of comorbidities and having SARS-CoV-2. Use of biologics was also not associated with a higher rate of SARS-CoV-2.	
**Campo-Slebi et al., 2021** (3)	Case series	Psoriatic on biologics	53	Most commonly hospitalization occurs when there is the presence of other comorbidities such as hypertension, obesity, and chronic liver disease. The hospitalization rate was lower in patients receiving biologic therapy (46.66% vs. 53.33%).	
**Conti et al., 2020** (4)	Case series	Psoriatic on biologics	4	All four cases remained in psoriasis remission as biologics were not discontinued. One (62M) was hospitalized and recovered fully within 1 month. One patient experienced symptoms (asthenia, anosmia, and ageusia) but recovered fully within 3 weeks. The final two patients tested positive but presented asymptomatic, despite both spending extended amounts of time with COVID-positive subjects.	
**Damiani et al., 2020** (5)	Case control	Psoriatic on biologics	1,193	Patients with psoriasis on biologic therapy displayed with a higher risk to be infected as well as hospitalized, but ICU admission and death did not vary from the general population.	Plaque psoriasis only, other types excluded
**Fougerousse et al., 2020** (6)	Multi-center cross-sectional	Psoriatic on biologics	1,418	No significant difference in severe cases of SARS-CoV-2 in patients on biologic therapy; 0.35% of the total cohort had to be hospitalized due to severe SARS-CoV-2 infection. From all patients hospitalized, 60% had more than two comorbidities.	13.8% discontinued biologic therapy during the pandemic
**Fulgecio-Barbarin et al., 2020** (7)	Retrospective observational	Psoriatic and frail	10	Frail patients (identified by the comorbidities) had a longer hospitalization as well due to having more severe SARS-CoV-2. Immunosuppressant treatment was suspended, although symptoms of infection were still present.	
**Galluzzo et al., 2020** (8)	Cross-sectional	Psoriatic on secukinumab	119	No cases of SARS-CoV-2 among any of the patients. Possible effective immune response in the presence of IL-17 inhibition.	
**Gisondi et al., 2020** (9)	Retrospective observational	Psoriatic on biologics and renal transplant on immunosuppressants	980 psoriasis and 243 renal transplant	Higher prevalence of comorbidities in patients with psoriasis and transplant patients compared with that in the general population. No patients with psoriasis required hospitalization, nor were there any deaths.	Patients with asymptomatic COVID excluded because of lack of testing
**Gisondi et al., 2020** (10)	Retrospective multi-center observational	Psoriatic	5,206	Four patients hospitalized all with comorbidities (renal failure, obesity and hypertension, and diabetes and hypertension). Overall, no significant risk of hospitalization and death compared with that in the general population.	
**Kartal et al., 2021** (11)	Multi-center observational	Psoriatic on biologics	1,827	Patients with psoriasis on biologic therapy do not have a higher risk of SARS-CoV-2 than the general population. Worsening of the psoriasis in most patients was due to drug non-adherence due to the pandemic. Receiving biologics was associated with a better disease course than those receiving conventional drugs.	
**Kridin et al., 2021** (12)	Retrospective cohort	Psoriatic on biologics	6,093	Patients on anti-TNFα showed a lower risk to hospitalization due to SARS-CoV-2 compared with patients on methotrexate, ustekinumab, and acitretin.	
**Lima et al., 2020** (13)	Retrospective cohort	Psoriatic	104	No increased rates of severe COVID in patients on systemic therapy.	Patients who have significant comorbidities have a decreased likelihood of being on systemic therapy. Propensity score was used to circumvent this.
**Mahil et al., 2020** (14)	Multi-center cross-sectional	Psoriatic on biologics	365	Biologic use was associated with lower risk of hospitalization due to SARS-CoV-2 infection; 21% of patients were hospitalized and most patients fully recovered from the infection; however, nine patients (2%) died, and all with at least one comorbidity.	71% received biologic therapy. Selection bias for biologics may be explained because of patients on biologics having less comorbidities.
**Pahalyats et al., 2021** (15)	Multi-center cross-sectional	Psoriatic on biologics	7,361	Patients on biologic therapy showed no difference in risk of infection with SARS-CoV-2 or mortality due to infection [Odds Ratio (OR): 0.88], and patients on anti-TNFα showed a lower incidence than the general population (OR: 0.69).	
**Piaserico et al., 2020** (16)	Retrospective observational	Psoriatic on biologics	1,830	No increased incidence rate (IR) or severity of SARS-CoV-2 in patients with psoriasis on biologics compared with that in the general population, despite a higher prevalence of comorbidities.	IR in patients with psoriasis was 9.7 per 10,000 person-months, whereas 11.5 per 10,000 person-months in the general population.
**Zitouni et al., 2022** (17)	Multi-center cross-sectional	Pediatric psoriatic	118	SAR-CoV-2 was just as prevalent in patients on biologic therapy as those on topical therapy; however, it often came with a longer course of disease in patients on biologics.	
**Ahmed et al., 2022** (18)	Cross-sectional	Psoriatic on biologics or apremilast	93	No difference in severity or susceptibility of SARS-CoV-2 in patients compared with that in the average ranges in Italy; seroprevalence was similar to that of the general population; 13% of patients tested positive for IgG SARS-CoV-2.	Patients with cardiovascular disease are at higher risk of contracting COVID-19
**Barrutia et al., 2021** (19)	Cross-sectional	Patients on biologics	99	Four patients had very high IgG (4.1%), similar to that of the general population of the same city (4.4%).	One patient was tested with COVID-19 incidentally without symptoms.
**Yendo et al., 2021** (20)	Cross-sectional	Psoriasis at risk of COVID-19	75	Twenty-four of the 75 patients with psoriasis were IgG positive (32%); seroprevalence of general population was 11%. No patients required hospitalization.	Outpatient clinic assists low-income population

**Table 2:** Efficacy and safety of the BNT162b2 vaccine in the population with psoriasis on biologic therapy.

Author, year	Study design	Type of patients	Number of patients	Doses	Results	Notes
**Zelini et al., 2022** (21)	Longitudinal cohort	Psoriatic on biologics	105	3	Patients on biologic therapy had lower titer of neutralizing antibodies and spike-specific T-cell immunity. Both control and patients on biologic therapy had a decline in immunity; however, a higher number of negative trimeric S IgG assays in patients on biologic therapy and no T-cell response. In addition, this group lagged in T-cell immunity after the booster dose.	
**Aikawa et al., 2022** (22)	Prospective cohort	Autoimmune rheumatic disease	164	4	A total of 17.1% of patients responded poorly to all four doses, and 95.1% of patients seroconverted after the fourth dose, compared with 66.4% after the third dose.	First three doses were Sinovac-CoronaVac inactivated vaccine.
**Al-Janabi et al., 2023** (23)	Prospective cohort	Inflammatory diseases on biologics	600	2	Patients with inflammatory mediated diseases on non-biologics had reduced odds of seroconversion, relative to those on mono biologic therapy. A total of 82.4% seroconverted after the first dose and 98.0% after the second dose. Only one patient on infliximab failed to seroconvert after the second dose.	
**Bieber et al., 2022** (24)	Retrospective cohort	Autoimmune rheumatic diseases on biologics	15,982	4	Patients vaccinated with the fourth dose had lower rates of infection, hospitalization, and death than those with only three doses.	
**Cristaudo et al., 2021** (25)	Cohort	Psoriatic on biologics	48	2	Significantly lower antibody titers in both doses in all patients receiving combination therapy than those on biologic monotherapy. Older age associated with a lower response to the vaccine. BMI did not affect the response to the vaccine.	28 with PsA, 43 BMI > 30. No flares after vaccination
**Graceffa et al., 2022** (26)	Prospective cohort	Psoriatic on biologics	45	3	No increased vulnerability to SARS-CoV-2 in patients with psoriasis or psoriatic arthritis. Third dose gave a 10-fold higher antibody titer after 4 weeks, than the same time after the second. No significant difference between titers of controls and patients.	
**Kvist-Hansen et al., 2023** (27)	Prospective cohort	Psoriatic on biologics and MTX	115	2	Lower IgG levels 1 month after second dose in patients receiving biologics compared with that in controls, with a faster waning of humoral immunity in patients receiving anti-TNFα agents. Similar results regarding cellular immunity after 6 months, with anti-TNFα patients performing worse than the other treatments. Positive correlation between cellular and humoral immunity in controls, with no correlation in patients on biologics.	Humoral immunity was measured for IgG with a cutoff of 225 AU/mL and IgA, with a cutoff of 100 AU/mL. Cellular immunity was measured by Interferon Gamma (IFNg) release (>200 mLU/mL)
**Lodde et al., 2023** (28)	Prospective single-center	Psoriatic on biologics	77	2	Three patients did not reach any IgG seroconversion, two of which were treated on biologics, neither of these patients had relevant comorbidities. There was a slightly reduced rate of seroconversion in patients receiving anti-TNFα and MTX compared with those receiving anti–IL-17 or anti–IL-12/23 biologics.	
**Mahil et al., 2022** (29)	Longitudinal cohort	Psoriatic on biologics or MTX	67	2	Worsening psoriasis in eight of the 67 patients with psoriasis. Second dose boosted spike-specific IgG in all patients, highest change in IgG from the first and second dosed in MTX patients, also with lower median titers. No change in T-cell response between two doses in patients on treatment. One-third of patients on treatment had no T-cell response.	Extended vaccine dose administration
**Marovt et al., 2022** (30)	Observational prospective	Psoriatic on biologics	32	2	No difference in the rate of seroconversion but significantly lower titers than the general population (1,023 vs. 3,055 BAU/mL), indicating the need for a booster shot. The type of biologic (anti-TNFα, anti–IL-12/23, anti–IL-17, and anti–IL-23) did not show any significant differences in response.	
**Megna et al., 2022** (31)	Prospective cohort	Psoriatic on biologics	44	2	No significant difference in antibody response or titers between patients with psoriasis and controls as well as between the different biologic groups.	Both BNT162b2 and mRNA-1273 were tested.
**Pavlotsky et al., 2021** (32)	Observational cohort	Psoriatic on biologics	51	2	There is 96% positive response to the vaccine; patients treated with IL-17 or IL-23 ( ± IL-23) inhibitors had antibody levels similar to controls but slightly higher than patients treated with anti-TNFα and MTX.	Two non-responders, both of which on anti-TNFα
**Sugihara et al., 2022** (33)	Prospective cohort	Rheumatic on biologics	123	2	Immunogenicity to the BNT162b2 vaccine was reduced in patients under immunosuppressive treatment (antibody titer was 108 U/mL); MTX particularly caused a significantly more reduced response.	Antibody titer for patients not on biologics was 927 U/mL. The average of healthy individuals was 741.6U/mL.
**Venerito et al., 2022** (34)	Prospective cohort	Psoriatic arthritis on anti-TNFα	40	1	Immunogenicity was not hindered by PsA or anti-TNFα.	
**Venerito et al., 2023** (35)	Observational prospective	Psoriatic arthritis on anti-TNFα	40	3	Patients with PsA had lower IgG levels than the controls. Booster dose restored IgG to the same level in both groups. No disease flares were recorded after booster dose.	

### 3.1 Course and severity of SARS-CoV-2 in patients with psoriasis on biologics

The definition of frailty concerns a wide range of patients and describes a clinical state in which there is an increase in an individual’s vulnerability to developing negative health-related events (including disability, hospitalizations, institutionalizations, and death) when exposed to endogenous or exogenous stressors (36, 37). According to the Italian Ministry of Health, patients on biologic therapy are considered frail, due to immunodeficiency from the inhibition of specific cytokines (38).

At the initial stages of the pandemic, Brazzelli et al. conducted an observational prevalence study over the phone with 180 patients with psoriasis on biologic therapy. Using a questionnaire on self-reported symptoms and SARS-CoV-2 positivity, they found that there was no increased incidence or severity of SARS-CoV-2 in this population. Furthermore, 18.3% of patients with psoriasis reported at least one symptom due to SARS-CoV-2, with no significant difference between patients on biologic therapies and those on other non-biologic therapies (1). With the same cohort of patients, the seroprevalence was assessed by Ahmed et al. It was found that 13% of the patients tested seropositive for SARS-CoV-2. The self-reported data being similar to the seroprevalence indicate a strong awareness of SARS-CoV-2 infection in these patients (1, 18).

Barrutia et al. observed that seroprevalence of SARS-CoV-2 in patients with psoriasis was in line with that of the general population (4.1% vs. 4.4%) (19). Another study by Yendo et al. found that the seroprevalence of SARS-CoV-2 in their cohort of patients with psoriasis of biologic therapy in Sao Paolo was higher than that in the general population (32% vs. 11%) (20). The authors suggest that the numbers are high due to the cohort of patients coming from a lower-income clinic, resulting in socioeconomic disparities, due to limited diagnostic tests, less access to education regarding the pandemic, and lack of contact tracing (20).

Conti et al. described four cases of SARS-CoV-2–positive patients with psoriasis on biologic therapy who fully recovered, only one of which required hospitalization (4). Galluzzo et al. reported that there was not a single SARS-CoV-2–positive case among their cohort of 119 patients with psoriasis on secukinumab, suggesting protective benefits from the biologic therapy (8).

A multitude of studies reported that the prevalence of COVID-19 infection rate and the length of recovery were higher in patients with psoriasis on biologic therapy when comorbidities were present. However, there was no significant difference in the rate of hospitalization or severe disease in psoriatic cohorts on biologic therapy compared with that in the general population (3, 7, 9–11, 17). Fougerousse et al. and Piaserico et al. reported lower rates of severe disease, despite higher rates of comorbidities within their cohorts patients with psoriasis on biologic therapy (6, 39). Mahil et al. found that, along with lower rates of hospitalization, patients on biologic therapy had lower rates of required mechanical ventilation and death (14).

Pahalyats et al. and Kridin et al. identified that there was no association between the use of biologics and SARS-CoV-2 infection and subsequent mortality. They also report that treatment with Tumour Necrosis Factor Alpha (TNFα) inhibitors showed lower infection rates (12, 15). Kridin et al. also noted that the patients on TNFα inhibitors showed a much better disease course compared with those on the other medications used as controls (12).

Lima et al. described that patients who have significant comorbidities have a decreased likelihood of being put on systemic therapy by their dermatologist, mainly due to the complications associated with biologic therapies. However, even when data are readjusted to overcome the variability, they found that patients with psoriasis on biologics were not at increased risk of severe SARS-CoV-2 infection (13).

Contradictorily, Baloghová et al. found that there was no relation between the incidence of SARS-CoV-2 and presence of comorbidities and the use of biologic therapy. The course of infection in most patients in their cohort was mild (2). Damiani et al. described that, within their cohort of patients with plaque psoriasis, the patients on biologic therapy had a higher risk of being infected and hospitalized. The risk of severe infection and death, however, was lower in this cohort (40). Mahil et al. also stated an insight regarding the correlation between biologics and lower rates of hospitalization that it may be due to selection bias; patients with multiple comorbidities are more likely to be put on non-specific therapy and only given biologic therapy in moderate-to-severe cases of psoriasis as well as the fact that patient on biologics being more prone to risk-mitigating behaviors, knowing that they have increased risk of infection due to the therapies (14).

### 3.2 Efficacy and safety of the BNT162b2 vaccine in the population with psoriasis on biologic therapy

Zelini et al. studied the humoral and cellular response to the BNT162b2 vaccine in patients with psoriasis on biologic therapy and found that a significant proportion of patients on biologics presented with a weakened humoral immune response. On administration of the booster dose, the humoral immunity of both groups was restored to previous levels, but 30.8% of patients on biologic therapy were not able to mount an appropriate cellular response after three doses, against all controls (21).

Regarding solely biologic therapy, several studies were conducted on patients with inflammatory disease on biologic therapy. Al-Janabi et al. reported that, after the second dose, seroconversion rose from 82.4% to 97.0% in patients with inflammatory disease on biologic therapy (23). Sugihara et al. found that the total antibody titer (IgG and IgM) was much lower in the patients on biologic therapy compared with those on non-biologic therapy and healthy controls (108.2 U/mL vs. 927 U/mL and 742.6 U/mL, respectively) (33).

However, several studies were also conducted analyzing the efficacy of the BNT162b2 vaccine in patients with psoriasis on biologic therapy. Most studies found that seroconversion was similar to that of the general population, with lower overall titers. Marovt et al. reported that, within their study, patients with psoriasis on biologics had 100% seroconversion rate. However, the titer levels were significantly lower compared with those in controls (30). Pavlotsky et al. discovered a 96% seroconversion rate [with Immunoglobulin G (IgG) titer levels similar to the controls] in the patients taking Interleukin (IL)-17 or -23 inhibitors and slightly higher than the titers of the patients on the other biologics studied (32). Cristaudo et al. described lower antibody titers in the population with psoriasis and that patients on monotherapy with biologic drugs showed higher IgG response rates than those on combination therapy (25). Lodde et al. found that, in their cohort, 97.0% of patients seroconverted. Two patients did not seroconvert; both patients were on infliximab, one on monotherapy and the other in conjunction with methotrexate (28).

Mahil et al. stated that all patients seroconverted after the second dose, and the IgG titers of patients on biologic therapy (mean titer: 1,816 U/mL) were not significantly different at the end of the second dose, relative to controls (mean titer: 2,749 U/mL). Responses against the alpha and delta variants in the patients on biologics were 97% and 36%, respectively, similar to the controls. Furthermore, after the second dose, only 71% of the patients on biologics showed a detectable T-cell response, which was significantly lower than the controls (100%) (29).

Megna et al. found no significant difference of effective antibody response between the control group and the psoriatic group; they also described the same trend in the antibody titer but a slightly higher average in the control group (41). Graceffa et al. also described that there was no significant difference in the humoral response to the BNT162b2 booster dose in patients with psoriasis compared with their control of healthy subjects, despite a decline in antibody titer 5 months after the second dose. Antibody titers 4 weeks after the booster dose were found to be 10-fold of those after the second dose. Patients on monotherapy had better humoral responses than those on combination therapy with methotrexate, but those patients still gave an effective response regardless. Conversely to other papers, they found no difference in response when comparing patients on TNFα inhibitors and anti–IL-17 monotherapies (26).

Venerito et al. conducted a study specifically to address the relation between immune response of the BNT162b2 vaccine and the use of TNFα inhibitors for psoriatic arthritis. The authors found no significant difference in immune response in these patients, with all patients presenting with a positive immune response (34). In a follow-up study studying the immunogenicity of the booster dose, given 4 months after the second dose, there was a higher decrease of IgG in patients with psoriasis compared with that in the control patients (85.2%, vs. 67.1%). It should be noted that, after the booster dose, the IgG titers returned to levels similar to those after the second dose, with patients with psoriasis having slightly lower levels, just as before (35).

Both cellular and humoral immunity against the vaccine were studied by Kvist-Hansen et al. The authors found that both types of immunity waned slowly within the 6 months in both the control and the patients on biologics, with most patients on biologic therapy having a positive humoral response (95%). The patients on TNFα inhibitors had the lowest humoral response with only 75.8%, whereas the anti–IL-17 group performed the best with a 100% response rate (27). Cellular responses were low in all patients on biologic therapy, especially in the TNFα inhibitor group, where only 44% responded cellularly. Overall, there was no correlation in humoral and cellular immunity in patients on biologics, with a positive correlation seen in the control group. The patients on TNFα inhibitors were found to have a bigger decrease of humoral and cellular immunity compared with the patients on other forms of biologics (with the patients on anti–IL-17 performing the best) (27).

The disproportion of the cellular and humoral response was studied by Hamm et al. and Cassaniti et al., who described the humoral and cellular responses in patients on immunosuppression after organ transplants; in both studies, the humoral and cellular responses are correlated in their decrease (42–44), signifying that the presence of the inflammatory disease may be an explanation for the findings with a lack of correlation between the two responses in their cohort of patients.

To date, no data on the efficacy of the fourth dose in patients with psoriasis on biologics are available in the literature. However, Aikawa et al. outlined the effect of the fourth dose of the BNT162b2 vaccine in patients with autoimmune rheumatic diseases on biologic therapy, 95.1% of patients showed an effective immune response, compared with 66.4% showing an effective immune response after three doses (22). Another study by Bieber et al. reported that the rates of infection, hospitalization, and death were lowest in their cohort of patients with autoimmune rheumatic disease who received the fourth dose, compared with those in the previous results after three doses (24).

## 4 Discussion

### 4.1 Course and severity of SARS-CoV-2 in patients with psoriasis on biologics

It can be suggested that the presence of psoriasis itself in patients is not correlated to an increase in the infection rate, hospitalization rate, or severity of disease due to SARS-CoV-2. The presence of heart disease, hypertension, and diabetes was studied extensively as there was a strong positive correlation to a worse prognosis of SARS-CoV-2 infection when comorbidities were present (39). However, with increased incidence of comorbidities within this population, there may be an increased risk of severe SARS-CoV-2 infection possibly requiring hospitalization (39, 45).

Regarding the relationship between biologic therapy and COVID-19 infection, it can be safely assumed that treatment of psoriasis using biologic drugs is not correlated with increased rates of infection. The National Psoriasis Foundation, in their guidelines concerning the COVID-19 pandemic and treatment of psoriasis, which was based on early studies and evidence during the pandemic, advises physicians to continue biologic therapy in patients with psoriasis, regardless of status of SARS-CoV-2 infection (45).

Along with the evidence of lower rates of hospitalizations, death rarely occurred within the population with psoriasis on biologics. The hidden advantages on the inhibition of certain cytokines within these biologic drugs may interfere with the pathogenic mechanism of SARS-CoV-2, allowing for a more favorable course of disease, as the cytokine storms that can be caused by the virion are prevented (46). In addition, IL-17 and TNFα inhibition have been hypothesized to be protective by preventing inappropriate inflammation due to cytokine storms (41, 46, 47). TNFα directly deteriorates the respiratory epithelium by producing inflammatory cytokines such as Granulocyte-Macrophage Colony Stimulating Factor (GM-CSF), IL-8, and intercellular adhesion molecules; worsening the course of the disease; and blocking TNF that has been shown to cause a rapid decline in the level of IL-6 and IL-1 in individuals with active inflammation (46, 48).

However, when prescribing anti-TNFα drugs, we should consider the presence of other immunosuppressive or corticosteroid drugs, the duration of treatments, and disease and comorbidities. All these factors are associated with elevated risk of infections (49, 50). Therefore, we should proceed with caution when evaluating risk and benefit of biologic therapy regarding SARS-CoV-2.

### 4.2 Efficacy and safety of the BNT162b2 vaccine in patients with psoriasis on biologics

From the data available, evidence shows that the seroconversion rate is not significantly lower in patients with psoriasis on biologic therapy after the BNT162b2 vaccine, compared with that in healthy individuals, suggesting that patients with psoriasis should be vaccinated regardless of treatment regime or status of disease. Among all studies reviewed, the totality of patients with psoriasis on biologic therapy seroconverted within three doses of BNT162b2, even if the response was negative following the first two doses. On the other hand, the cellular response to the vaccine was weaker compared with the humoral response, with a large number of patients not being able to mount an appropriate cellular response within three doses of the BNT162b2 vaccine. The presence of effective, albeit weaker, responses to the vaccine may prove that patients with psoriasis on biologic therapy may benefit from a tighter vaccination schedule and the booster dose.

Regarding the fourth BNT162b2 dose in these patients, evidence still remains inconclusive. The use of a fourth dose in patients who are non-responders may potentially allow for the activation of an immune response, even if activation was not achieved from the three previous doses (24, 51). A fourth dose may allow for a prolonged immune response in those that responded well to previous doses but had decreased time of effectiveness (52).

## 5 Conclusion

It is clear that patients on biologic therapy have to be treated in a more personalized way, despite having an infection rate comparable with the general population. There are increased rates of comorbidities in these patients due to the multiple inflammatory mechanisms associated with psoriasis (53, 54).

The increased rates of comorbidities put these patients at an elevated risk of severe infection leading to hospitalization (14, 39, 55). It can be argued that this makes the patient frail; despite not being more vulnerable to SARS-CoV-2 infection than the general population, they have a higher risk of severe disease (54).

However, the use of biologic therapy has been suggested to have protective mechanisms, inhibiting pathologic cytokine pathways associated with the cytokine storm that occurs due to SARS-CoV-2. The inhibition of cytokines such as TNFα and IL-17, in particular, has been shown to reduce the levels of other cytokines and to reduce the active SARS-CoV-2 infection (46, 48, 56). This may play a role in the decreased rates of severe disease and hospitalizations due to SARS-CoV-2 infection in patients on biologic therapy. However, we must always approach these cases with caution as the use of biologic therapy may increase the risk of other opportunistic infections (57).

However, because of the smaller sample sizes and variations in treatment regimens within literature, it is difficult to provide a strong conclusion. There is still a need for ongoing studies that encompass the infection rates and immune responses in patients on biologic therapy during SARS-CoV-2 infection as well as follow-up studies on the maintenance of response post-vaccination and whether this has a real impact on the frequency of infections.

## Author contributions

JR: Conceptualization, Data curation, Investigation, Methodology, Writing – original draft, Writing – review & editing. MV: Conceptualization, Data curation, Investigation, Writing – original draft, Writing – review & editing. EI: Conceptualization, Data curation, Investigation, Writing – review & editing. AB: Conceptualization, Data curation, Investigation, Writing – review & editing. SB: Conceptualization, Data curation, Investigation, Writing – original draft, Writing – review & editing. VB: Conceptualization, Data curation, Investigation, Writing – original draft, Writing – review & editing, Formal Analysis, Supervision, Methodology.
